# Predictive IDH Genotyping Based on the Evaluation of Spatial Metabolic Heterogeneity by Compartmental Uptake Characteristics in Preoperative Glioma Using ^18^F-FET PET

**DOI:** 10.2967/jnumed.123.265642

**Published:** 2023-11

**Authors:** Johannes Lohmeier, Helena Radbruch, Winfried Brenner, Bernd Hamm, Anna Tietze, Marcus R. Makowski

**Affiliations:** 1 Department of Radiology, Charité–Universitätsmedizin Berlin, Berlin, Germany;; 2 Department of Neuropathology, Charité–Universitätsmedizin Berlin, Berlin, Germany;; 3 Department of Nuclear Medicine, Charité–Universitätsmedizin Berlin, Berlin, Germany;; 4 Institute of Neuroradiology, Charité–Universitätsmedizin Berlin, Berlin, Germany; and; 5 Department of Radiology, Technical University Munich, Munich, Germany

**Keywords:** ^18^F-FET PET/MRI, amino acid metabolism, IDH genotyping, biomarker, spatial metabolic heterogeneity

## Abstract

Molecular markers are of increasing importance for classifying, treating, and determining the prognosis for central nervous system tumors. Isocitrate dehydrogenase (IDH) is a critical regulator of glucose and amino acid metabolism. Our objective was to investigate metabolic reprogramming of glioma using compartmental uptake (CU) characteristics in *O*-(2-^18^F-fluoroethyl)-l-tyrosine (FET) PET and to evaluate its diagnostic potential for IDH genotyping. **Methods:** Between 2017 and 2022, patients with confirmed glioma were preoperatively investigated using static ^18^F-FET PET. Metabolic tumor volume (MTV), MTV for 60%–100% uptake (MTV_60_), and T2-weighted and contrast-enhancing lesion volumes were automatically segmented using U-Net neural architecture and isocontouring. Volume intersections were determined using the Dice coefficient. Uptake characteristics were determined for metabolically defined compartments (central [80%–100%] and peripheral [60%–75%] areas of ^18^F-FET uptake). CU ratio was defined as the fraction between the peripheral and central compartments. Mean target-to-background ratio was calculated. Comparisons were performed using parametric and nonparametric tests. Receiver-operating-characteristic curves, regression, and correlation were used for statistical analysis. **Results:** In total, 52 participants (male, 27, female, 25; mean age ± SD, 51 ± 16 y) were evaluated. MTV_60_ was greater and distinct from contrast-enhancing lesion volume (*P* = 0.046). IDH-mutated tumors presented a greater volumetric CU ratio and SUV CU ratio than IDH wild-type tumors (*P* < 0.05). Volumetric CU ratio determined IDH genotype with excellent diagnostic performance (area under the curve [AUC], 0.88; *P* < 0.001) at more than 5.49 (sensitivity, 86%, specificity, 90%), because IDH-mutated tumors presented a greater peripheral metabolic compartment than IDH wild-type tumors (*P* = 0.045). MTV_60_ and MTV were not suitable for IDH classification (*P* > 0.05). SUV CU ratio (AUC, 0.72; *P* = 0.005) and target-to-background ratio (AUC, 0.68; *P* = 0.016) achieved modest diagnostic performance—inferior to the volumetric CU ratio. Furthermore, the classification of loss of heterozygosity of chromosomes 1p and 19q (AUC, 0.75; *P* = 0.019), MGMT promoter methylation (AUC, 0.70; *P* = 0.011), and ATRX loss (AUC, 0.73; *P* = 0.004) by amino acid PET was evaluated. **Conclusion:** We proposed parametric ^18^F-FET PET as a noninvasive metabolic biomarker for the evaluation of CU characteristics, which differentiated IDH genotype with excellent diagnostic performance, establishing a critical association between spatial metabolic heterogeneity, mitochondrial tricarboxylic acid cycle, and genomic features with critical implications for clinical management and the diagnostic workup of patients with central nervous system cancer.

## Visual Abstract

**Figure:**
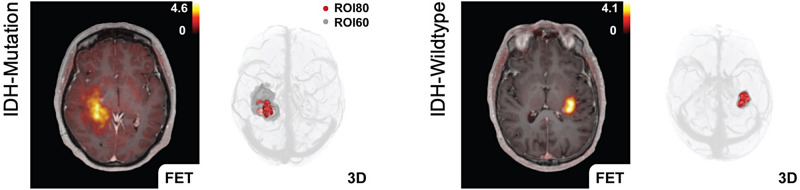

Gliomas are the most common primary malignant neoplasms of the central nervous system (CNS) in adults and comprise a large spectrum of molecular subtypes with intricate pathophysiology. Molecular stratification is essential for diagnosis, treatment planning, and individual prognosis. In recent years, the World Health Organization (WHO) classification of tumors of the CNS has undergone several updates (1) introducing several important changes, such as the incorporation of molecular and genetic information. One important molecular marker that has gained significant attention is the mutation status of the isocitrate dehydrogenase (IDH) gene (2–4). IDH gene mutations play a central role in glioma pathophysiology, occurring early in the glioma genesis and characterizing a group of tumors that is molecularly distinct from primary glioblastoma. Because IDH mutations are associated with a more favorable prognosis (5), the IDH genotype has become a central feature in the diagnosis and management of patients with CNS cancer. In recent years, considerable progress in understanding the molecular mechanisms and pathophysiology underlying IDH mutations has been made. IDH genes encode a key enzyme in the tricarboxylic acid cycle, which is a central cellular pathway for energy production. When IDH genes are altered, a profound disruption in the tricarboxylic acid cycle with dysregulation of the amino acid metabolism is induced—a hallmark of CNS cancer—which is leveraged for bioenergetic processes and protein synthesis. By a gain-of-function mutation, the physiologic conversion of isocitrate to α-ketoglutarate, an important intermediate metabolite in the Krebs cycle, is inhibited, whereas the production of d-2-hydroxyglutarate is propagated. High levels of the oncometabolite d-2-hydroxyglutarate mediate global DNA and histone hypermethylation, impairment of DNA break repair mechanisms, and a decrease in hypoxia-inducible factors through competitive inhibition of tumor suppressors in the α-ketoglutarate–dependent dioxygenase family that contribute to glioma pathogenesis and progression through alteration of cellular differentiation, proliferation, and gene expression (2*,*^3^).

In the surgical management of patients with preoperative IDH-mutated glioma, supramaximal resection was shown to improve overall survival (5–8). However, the IDH genotype is typically unknown before surgery, and a preceding (stereotactic or open) biopsy involves the hazards of perioperative complications. To date, there are few reliable means for noninvasive and predictive genotyping of IDH mutation status in clinical practice (5).

On the basis of the hypothesis that specific genetic alterations are linked with distinct metabolic phenotypes, we introduced the compartmental uptake (CU) ratio as a noninvasive metabolic imaging biomarker characterizing the spatially heterogeneous glioma metabolism by differentiating the metabolic tumor core from its periphery. Predictive genotyping of IDH mutation status was then investigated using *O*-(2-^18^F-fluoroethyl)-l-tyrosine (FET) PET, as an established marker for amino acid metabolism, in a patient cohort with preoperative glioma.

## MATERIALS AND METHODS

### Study Design and Patients

**FIGURE 1.**
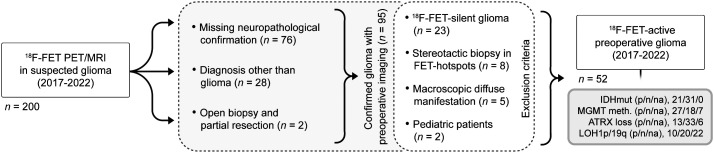
Study selection flowchart. ATRX = α-thalassemia/mental retardation syndrome, X-linked; MGMT = *O*^6^-methylguanine DNA methyltransferase. p/n/na = positive/negative/not applicable.

This retrospective clinical cohort study was conducted according to the principles of the Helsinki Declaration. Approval from the institutional ethics board was obtained (EA2/019/23). Informed consent was obtained from all participants. From 200 consecutive suspected-glioma patients evaluated using a simultaneous ^18^F-FET PET/MRI approach between 2017 and 2022, 52 participants with hybrid imaging before resection (Fig. 1) were included according to the eligibility criteria.

### Neuropathologic Analysis

The molecular status of IDH mutation (IDH-mutated or wild-type), 1p/19q codeletion (loss of heterozygosity of chromosomes 1p and 19q [LOH1p/19q]–positive, codeleted; LOH1p/19q-negative, nondeleted), MGMT promoter methylation (MGMT-positive, methylated; MGMT-negative, unmethylated), and ATRX loss (ATRX-positive, deficient; ATRX-negative, retention) were determined from formalin-fixed paraffin-embedded tissue specimens during routine diagnostic workup procedures using fluorescence in situ hybridization analysis, pyrosequencing, EPIC DNA methylation arrays (Illumina), or immunostainings according to the requirements of the WHO classification of tumors of the CNS (1). When pyrosequencing of MGMT promoter methylation was used, a cutoff of 10% was defined to classify MGMT methylated versus unmethylated cases, a cutoff that is commonly applied and has been validated for routine clinical diagnostics (9). Gliomas were classified using the 2021 WHO classification (1) according to the molecular data available at that time point.

### PET/MRI Acquisition

Simultaneous PET/MRI was performed on a Magnetom Biograph mMR scanner (Siemens Healthcare) with an averaged axial spatial resolution of 6 mm in full width at half maximum, which was determined in a 3-dimensional Hoffman brain phantom measurement (ordered-subsets expectation maximization, 3 iterations and 21 subsets, postfiltering by a 3-dimensional gaussian kernel of 3 mm in full width at half maximum, as in patient data) following the method by Joshi et al. (10). PET and clinical high-field (3-T) MRI were performed in list mode for up to 60 min after intravenous administration of ^18^F-FET (mean  ±  SD, 163  ±  23 MBq; 180-MBq standard dose and individually calculated dose for body weight  <  60 kg). Fasting for at least 4 h before PET acquisition was recommended. A gadolinium-based contrast agent (Gadovist; Bayer Pharma AG) was administered according to the patient’s total body weight (0.1 mmol/kg). The MRI acquisition protocol included a transversal T1-weighted ultrashort echo time sequence for attenuation and scatter correction, a T2-weighted sequence (repetition time/echo time,  5,320/88 ms; matrix size,  230 × 230 × 230; voxel size,  0.4 × 0.4 × 3.0 mm), and a postcontrast T1-weighted magnetization-prepared rapid gradient echo sequence (repetition time/echo time/inversion time,  2,400/2.26/900 ms; flip angle,  8°; matrix size,  256 × 256 × 256; voxel size,  1.0 × 1.0  × 1.0 mm; thickness,  1 mm; slices,  192). Vendor-based attenuation correction (software versions MR B20P and MR E11P) using ultrashort echo time was performed. The PET acquisition was reconstructed into transaxial slices using an iterative ordered-subset expectation maximization algorithm (ordered-subsets expectation maximization, 3 iterations and 21 subsets; matrix size,  344 × 344 × 127; voxel size,  1.0  × 1.0  × 2.3 mm; gaussian filter,  3 mm). Emission data were corrected for decay, randoms, dead time, scatter, and attenuation.

### PET and MRI Analysis

Quantitative analysis was performed using OsiriX MD 12 (Pixmeo SARL). Contrast-enhancing and T2-weighted lesion volumes were determined in an automated manner using an attention-based U-Net architecture with postcontrast T1-weighted magnetization-prepared rapid gradient echo and T2-weighted/fluid-attenuated inversion recovery images as input (11). When applicable, regions of interest (ROIs) of T2-weighted and contrast-enhancing lesion volumes were slightly adapted. ^18^F-FET uptake was measured in an automated manner using isocontouring based on attenuation-corrected ^18^F-FET tracer uptake, yielding an uptake-based total (60%–100%, MTV_60_), peripheral (60%–75%, ROI_60_), and central (80%–100%, ROI_80_) metabolic compartment (thresholds were iteratively determined in a pilot experiment, differentiating uptake pattern into a central and peripheral compartment based on visual assessment). Segmentations were marginally adapted to exclude large intracranial blood vessels (when applicable). For definition of metabolic tumor volume, both a percentage method (60%–100%, MTV_60_) and an absolute threshold method (1.8 times the mean uptake of the healthy contralateral background, MTV) were used. CU ratio was defined as the fraction between ROI_60_ and ROI_80_, yielding a volumetric CU ratio for the uptake-based volume or SUV CU ratio for the mean SUV. Mean target-to-background ratio was determined from SUV_mean_ on the basis of a 3-dimensional VOI (ROI_80_) from an ^18^F-FET–active lesion compared with the mean unaffected contralateral background to account for nonspecific and regional uptake behavior. In multifocal tumor manifestations, the most prominent ^18^F-FET–active lesion was chosen as the target lesion. Mean background tracer uptake was computed from a contralateral 2-dimensional ROI of similar size in unaffected brain tissue on a representative slice with the highest mean uptake within the tumor volume. Three-dimensional ROIs from magnetization-prepared rapid gradient echo and ^18^F-FET PET were then transformed to a structural T2-weighted image using rigid deformation (ANTs, version 2.3.4).

### Statistics

Statistical analysis was performed using Prism version 9 (GraphPad Software) and MedCalc version 20.104 (MedCalc Software Ltd.). Mann–Whitney *U* (2-tailed) tests (with Holm-Šídák multiple-comparison testing) were used for comparisons between 2 groups. Wilcoxon signed-rank (1-tailed) testing was used for matched pairs. Kruskal–Wallis testing (with Dunn multiple-comparison testing) was used for comparisons among 3 groups. Receiver-operating-characteristic analysis was performed using the DeLong method reporting area under the curve (AUC), 95% CIs, and *P* value. Sensitivity and specificity were reported for the best cutoff point independent of the prevalence determined using the Youden index. Logistic regression was used to model binary outcome (predictive accuracy based on a *P* value cutoff of 0.5). Measurements were correlated and evaluated using the nonparametric Spearman correlation coefficient (2-tailed). Intersections between volumes were computed on the basis of the Dice coefficient (Eq. 1) and the Jaccard index. In all tests, a *P* value of less than 0.05 was considered statistically significant.

$$2\times \frac{\left|X\cap Y\right|}{\left|X\right|+\left|Y\right|}$$ Eq. 1

## RESULTS

### Patient Population

Overall, the patient cohort comprised 52 participants (male, 27, female, 25; age, mean ± SD, 51 ± 16 y) with preoperative ^18^F-FET–active glioma (high-grade glioma, 45; low-grade glioma, 5; not applicable, 2) and available neuropathologic classification (Fig. 1; Table 1). Molecular data of IDH mutation (IDH-mutated, 40%, IDH wild-type, 60%), MGMT promoter methylation (MGMT-positive, 52%; MGMT-negative, 35%; unknown, 13%), LOH1p/19q (LOH1p/19q-positive, 19.2%; LOH1p/19q-negative, 38.5%; unknown, 42.3%), and ATRX loss (ATRX-positive, 25%; ATRX-negative, 63%; unknown,12%) were determined. CNS WHO grade 4 tumors were predominantly IDH wild-type (93%), whereas most WHO CNS grade 2–3 tumors were IDH-mutated (86%). IDH-mutated gliomas included both astrocytoma (48%) and oligodendroglioma (52%). The study cohort comprised a case of diffuse pediatric-type high-grade glioma in an adult patient. A detailed overview of the molecular stratification is available in Supplemental Table 1 (supplemental materials are available at http://jnm.snmjournals.org).

**TABLE 1.** Characteristics of Patient Cohort

Characteristic	Data
Participants	52
Age (mean ± SD in y)	51 ± 16
Sex	
Male	27
Female	25
IDH mutation status	
Positive	21
Negative	31
Not applicable	0
MGMT promoter methylation status	
Positive	27
Negative	18
Not applicable	7
ATRX loss	
Positive	13
Negative	33
Not applicable	6
LOH1p/19q	
Positive	10
Negative	20
Not applicable	22

Data are number, except for age.

### Comparison of Volumetric Attributes in Preoperative Glioma

**FIGURE 2.**
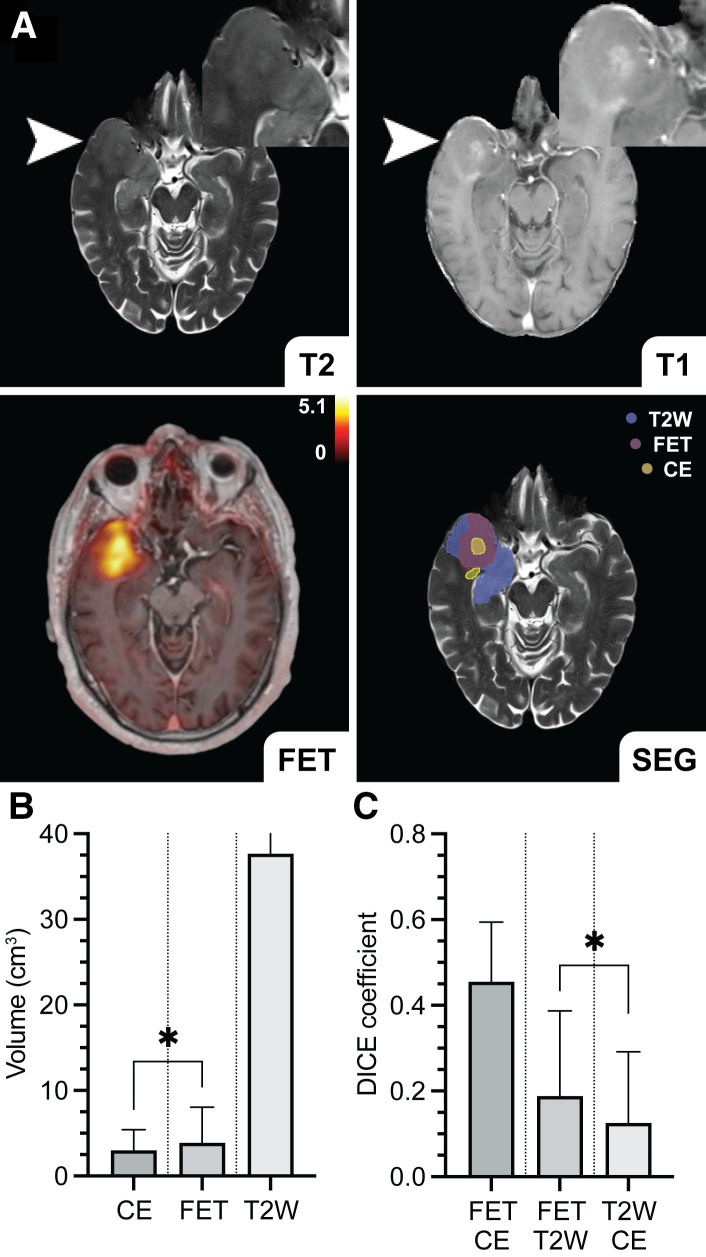
Volumetric analysis in multimodal ^18^F-FET PET/contrast-enhanced MRI. (A) Automated segmentation based on ^18^F-FET PET, contrast-enhancing, and T2-weighted lesion volume in multimodal dataset of patient with glioblastoma IDH wild-type (CNS WHO grade 4, MGMT-negative, ATRX-negative; arrowheads) is illustrated. (B and C) In intrasubject comparison, MTV_60_ was greater than contrast-enhancing lesion volume (*P* = 0.046, *n* = 30). Furthermore, intersection or overlap between T2-weighted lesion volume and MTV_60_ was greater than its overlap with contrast-enhanced MRI (*P* = 0.038, *n* = 30). Dice coefficients presented low to moderate degree of overlap or similarity between respective volumes, although MTV_60_ and contrast-enhancing lesion volume showed highest concordance (C). Data are median  ±  IQR. *P  <  0.05. SEG = segmentation.

MTV_60_ from ^18^F-FET PET was greater than contrast-enhancing lesion volume (median, 3.87 cm^3^ [interquartile range (IQR), 5.47] vs. 3.00 cm^3^ [IQR, 4.66], *P* = 0.046, *n* = 30, Kendall W statistic, −165) in intrasubject comparison (Fig. 2). Correspondingly, the intersection of T2-weighted lesion volume with MTV_60_ was greater than its overlap with contrast-enhancing lesion volume (median, 0.189 [IQR, 0.327] vs. 0.125 [IQR, 0.262], *P* = 0.038, *n* = 30, Kendall W statistic, −173) as determined by the Dice coefficient. T2-weighted lesion volume showed the greatest dimensions and variability because of perilesional edema and diffuse infiltration. Volume intersections between MTV_60_, contrast-enhancing lesion volume, and T2-weighted lesion volume presented a low to moderate degree of overlap or similarity (Fig. 2C), although MTV_60_ and contrast-enhancing lesion volume (Dice coefficient; median, 0.455 [IQR, 0.372]) presented the greatest concordance and were weakly correlated (Spearman rank correlation coefficient, 0.38; *P* = 0.038).

**TABLE 2.** Diagnostic Measures from ^18^F-FET PET for IDH Classification

	Mann–Whitney *U* test			ROC					
Metric	Adjusted *P*	Mean rank difference	*U*	*P*	AUC ± SE	95% CI	Threshold	Sensitivity (%)	Specificity (%)
Volumetric CU ratio	<0.001	−19.93	76	<0.001	0.88 ± 0.05	0.76–0.96	>5.49	86	90
MTV_60_	>0.05	−6.430	245	>0.05	0.62 ± 0.08	0.48–0.75	—		
MTV	>0.05	−1.717	304	>0.05	0.53 ± 0.09	0.39–0.67	—		
SUV CU ratio	0.032	−11.22	185	0.005	0.72 ± 0.08	0.57–0.83	>0.77	76	71
Target-to-background ratio	>0.05	9.386	208	0.016	0.68 ± 0.07	0.54–0.80	≤2.73	76	58

ROC = receiver operating characteristic.

### Association Between Molecular Status and CU Characteristics

**FIGURE 3.**
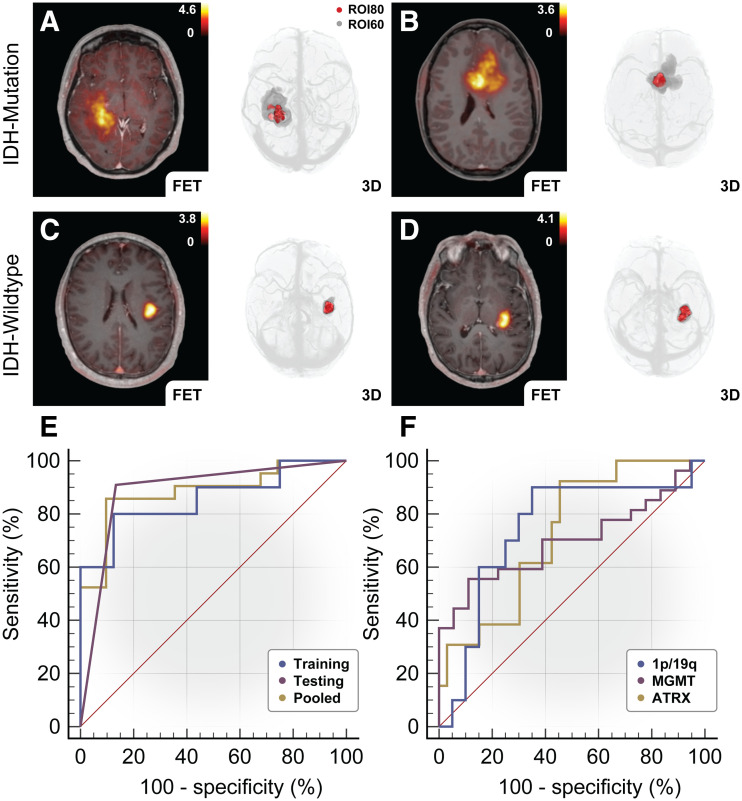
Evaluation of volumetric CU ratio for differentiation between IDH mutation status. (A and B) Representative cases of IDH-mutated high-grade astrocytoma (CNS WHO grade 3) (A) and IDH-mutated low-grade oligodendroglioma 1p/19q codeleted (CNS WHO grade 2) (B) are illustrated with ^18^F-FET PET/magnetization-prepared rapid gradient echo fusion image and 3-dimensional rendering, respectively, demonstrating increased volumetric CU ratio (ROI_60_/ROI_80_). (C and D) In contrast, IDH wild-type presented low volumetric CU ratio as shown in glioblastoma IDH wild-type (CNS WHO grade 4). (E and F) Genotyping of IDH mutation status using CU ratio based on receiver-operating-characteristic analysis: volumetric CU ratio demonstrated robust diagnostic performance for differentiation between IDH-mutated and IDH wild-type tumors in independent model-building (training) dataset (AUC, 0.86 ± 0.09; *P* < 0.001; 95% CI, 0.66–0.96; IDH wild-type, 16; IDH-mutated, 10) with threshold > 5.43 (sensitivity, 80%, specificity, 88%) and evaluation (testing) dataset (AUC, 0.89 ± 0.06; *P* < 0.001; 95% CI, 0.70–0.98; IDH wild-type, 15; IDH-mutated, 11) as well as in entire study cohort (AUC, 0.88 ± 0.05; *P* < 0.001; 95% CI, 0.76–0.96) (E); in addition, differentiation between LOH1p/19q status, MGMT promoter methylation status, and ATRX loss was evaluated, presenting moderate diagnostic performance (*P* < 0.05) (F).

IDH-mutated tumors presented a greater volumetric CU ratio (median, 7.84 [IQR, 5.20, *n* = 21] vs. 3.92 [IQR, 2.32, *n* = 31], *P* < 0.001, *U* = 76) and SUV CU ratio (median, 0.781 [IQR, 0.024, *n* = 21] vs. 0.768 [IQR, 0.022, *n* = 31], *P* = 0.032, *U* = 185) than IDH wild-type, as demonstrated in Table 2. For further evaluation of the volumetric CU ratio, the clinical cohort was randomly split into an independent model-building-and-assessment dataset based on neuropathologic IDH classification. Excellent diagnostic performance for the differentiation between IDH genotype (Fig. 3E) was apparent in the model-building dataset (AUC ± SE, 0.86 ± 0.09; *P* < 0.001; 95% CI, 0.66–0.96; IDH wild-type, 16; IDH-mutated, 10), which determined an optimal threshold of 5.43 (sensitivity, 80%; specificity, 88%; accuracy, 81%) based on the Youden index. This was then confirmed in the independent evaluation dataset (AUC ± SE, 0.89 ± 0.06; *P* < 0.001; 95% CI, 0.70–0.98; IDH wild-type, 15; IDH-mutated, 11) applying the previously determined threshold of 5.43 for IDH-mutated (sensitivity, 91%; specificity, 87%; accuracy, 88%) with similar results for the entire cohort (AUC ± SE, 0.88 ± 0.05; *P* < 0.001; 95% CI, 0.76–0.96). As shown in Figures 3A–3D, IDH-mutated tumors presented a greater peripheral metabolic compartment (corresponding to ROI_60_) than IDH wild-type (median, 5.33 [IQR, 7.47, *n* = 21] vs. 2.78 [IQR, 4.94, *n* = 31], *P* = 0.045, *U* = 218), whereas no difference for the central metabolic compartment was apparent (*P* > 0.05). MTV_60_ and MTV were not suitable for IDH classification (*P* > 0.05). A low correlation between volumetric CU ratio was observed for MTV_60_ (Spearman rank correlation coefficient, 0.356; *P* = 0.01) but not for MTV (*P* > 0.05). With a more conservative thresholding method, MTV_60_ underestimated the total tumor volume compared with the clinically established MTV (median, 4.95 cm^3^ [IQR, 7.46] vs. 7.75 cm^3^ [IQR, 10.77]; *P* = 0.018; *n* = 52; Kendall W statistic, 460). SUV CU ratio (AUC ± SE, 0.72 ± 0.08; *P* = 0.005; accuracy, 67%) and target-to-background ratio (AUC ± SE, 0.68 ± 0.07; *P* = 0.016; accuracy, 63%) achieved modest diagnostic power, although the performance of target-to-background ratio was inferior to that of volumetric CU ratio (difference in AUC ± SE, 0.203 ± 0.080; *P* = 0.01).

Comparisons between IDH-mutated oligodendroglioma (*n* = 11), IDH-mutated astrocytoma (*n* = 8), and IDH wild-type glioblastoma (*n* = 25) with regard to target-to-background ratio (*P* > 0.05; Kruskal–Wallis test, 4.125), SUV CU ratio (*P* = 0.028; Kruskal–Wallis test, 7.163; adjusted *P* > 0.05), and volumetric CU ratio (*P* < 0.001; Kruskal–Wallis test, 18.55) demonstrated that IDH wild-type glioblastoma presented lower volumetric CU ratios than IDH-mutated oligodendroglioma (median, 3.88 [IQR, 1.97] vs. 8.75 [IQR, 7.21]; *P* < 0.001; mean rank difference, 17.40) with a classification threshold (AUC ± SE, 0.88 ± 0.07; *P* < 0.001; 95% CI, 0.73–0.96; sensitivity, 82%; specificity 88%; accuracy, 81%) at 5.26 (based on the Youden index) and IDH-mutated astrocytoma (median, 3.88 [IQR, 1.97] vs. 7.89 [IQR, 2.17]; *P* = 0.007; mean rank difference, 16.01) with the same classification threshold (AUC ± SE, 0.89 ± 0.09; *P* < 0.001; 95% CI, 0.73–0.97; sensitivity, 88%; specificity 88%; accuracy, 82%) at 5.26 (based on the Youden index). No differences between IDH-mutated astrocytoma and oligodendroglioma were apparent (*P* > 0.05). Supplemental Table 2 provides further details.

Molecular classification of LOH1p/19q (AUC ± SE, 0.75 ± 0.11; *P* = 0.019; accuracy, 67%), MGMT promoter methylation (AUC ± SE, 0.70 ± 0.08; *P* = 0.011; accuracy, 64%), and ATRX loss (AUC ± SE, 0.73 ± 0.08; *P* = 0.004; accuracy, 74%) were additionally evaluated using volumetric CU ratio (Fig. 3F).

## DISCUSSION

We introduced a noninvasive metabolic imaging biomarker for the assessment of metabolic reprogramming in gliomas and demonstrated its diagnostic potential for the predictive genotyping of IDH mutation status by characterizing the spatially heterogeneous amino acid metabolism in a patient cohort with preoperative glioma. First, we showed that the MTV_60_ in ^18^F-FET PET is distinct from contrast-enhanced MRI, which is the clinical standard for the initial diagnosis, biopsy targeting, and surveillance of brain tumors. Furthermore, MTV_60_ presented greater dimensions than contrast-enhanced MRI, which is known to underestimate tumor margins because of diffuse infiltration beyond areas of blood–brain barrier impairment (12*,*^13^). Exploiting the cancer amino acid metabolism, we proposed the volumetric CU ratio as a biologic determinant for the assessment of CU characteristics, which determined IDH mutation status in this cohort of treatment-naïve glioma patients with excellent diagnostic accuracy, suggesting a central role for noninvasive genotyping before surgical intervention.

Gene expression in glioma is known to be spatially distinct, for example, presenting differential upregulation of tyrosine aminotransferase—where increased expression in the tumor core as opposed to the periphery was reported—and a corresponding activation of the tyrosine metabolism (14). Because of this differential expression and metabolism, the IDH genotype, which is a critical regulator of both glucose and amino acid metabolism (15), was determined in an indirect bottom-up approach in the current study. Interestingly, LOH1p/19q, MGMT promoter methylation, and ATRX loss were differentiated by spatial metabolic patterns—although diagnostic performance was moderate—suggesting indirect associations. Similar to results from previous metabolic imaging studies (16–18), the averaged ^18^F-FET uptake differentiated IDH genotype with modest diagnostic performance—inferior to the volumetric CU ratio. MTV_60_ and MTV were not suitable for differentiation between IDH genotypes, but a weak correlation between MTV_60_ and volumetric CU ratio was observed. CU characteristics may also be determined as a ratio of total (summed) uptake (instead of volume) or may be accessible from histogram analysis. Although IDH-mutated astrocytoma and oligodendroglioma are considered distinct tumor entities, both could be independently differentiated from IDH wild-type glioblastoma at the same threshold, further suggesting that the volumetric CU ratio reflects metabolic reprogramming dependent on the IDH genotype. We could not observe increased uptake in IDH-mutated oligodendroglioma compared with IDH-mutated astrocytoma, as suggested in previous studies (17*,*^19^)—likely because of the relatively low number of these tumors in the cohort. When metabolic compartments were compared between IDH-mutated and IDH wild-type glioma, only the peripheral compartment was increased in IDH-mutated tumors, which corresponded to a visually apparent heterogeneous uptake observed in some tumors.

### IDH Genotyping Using Advanced Imaging

Determination of the IDH mutation status from conventional MRI is difficult, although IDH-mutated, LOH1p/19q-negative low-grade gliomas were reported to present T2-weighted or fluid-attenuated inversion recovery mismatch (20), which has low sensitivity (42%) but high specificity (nearly 100%) for this distinct entity. It must, however, be noted that dysembryoplastic neuroepithelial tumors were also reported to exhibit this imaging characteristic (21). Previous research efforts focused predominantly on measurement of d-2-hydroxyglutarate using MR spectroscopy (22–26), but its clinical implementation has been limited by low spatial resolution (voxel size > 1 cm^3^) and signal-to-noise ratio, as well as the propensity to artifacts, particularly in the posttreatment setting. Moreover, d-2-hydroxyglutarate MR spectroscopy requires off-line postprocessing, currently impeding more widespread implementation. Of note is that single-voxel MR spectroscopy depends on the accuracy of the voxel placement in often heterogeneous tumors. Diffusion MRI was shown to correspond to the IDH genotype in CNS WHO 2 and 3 gliomas (27); however, modest diagnostic power for the differentiation of IDH mutation status was apparent using both single- and multiple-shell imaging. A study by Lohmann et al. (28) using ^18^F-FET PET radiomics suggested that the combined biparametric analysis of conventional uptake parameters with additional textural features can achieve a similar diagnostic accuracy (as reported here); nonetheless, textural feature analysis is a complex and time-demanding approach, which suffers from well-known issues of restricted generalizability, overfitting, or other methodologic flaws (29). Experimental advanced imaging techniques, such as chemical exchange saturation transfer (30) or hyperpolarized ^13^C-MRI (31), were also demonstrated to show correspondence to the IDH genotype, but clinical implementation is currently challenging. In contrast, the current study’s approach demonstrated robust IDH classification based on static ^18^F-FET PET without the need for complex analysis.

### Clinical Relevance of Biomarker-Driven IDH Classification

The IDH genotype is a clinically important marker for molecular targeting, surgical planning, and individual prognosis (5). With the paradigm shift to molecular markers in clinical management of CNS cancer, there is a great clinically unmet need for noninvasive biomarker-driven classification. In clinical settings where limited tissue specimens are obtained, such as in stereotactic minimally invasive MRI-guided laser ablation or laser interstitial thermal therapy (32), an additional clinical benefit is expected. Molecular stratification before surgical intervention provides opportunities for more effective individualized neoadjuvant therapeutic strategies, an important topic in multimodal cytoreductive therapy because IDH-mutated gliomas are associated with better outcomes from radiochemotherapy (2*,*^3^). Furthermore, an imaging biomarker–driven classification aids the identification of patients with an increased risk of recurrence, allowing for earlier and more aggressive treatment regimens.

### Limitations

General conclusions should be drawn with caution because of the retrospective nature of this study. Further research with larger multicenter cohorts (with different scanners and reconstruction settings) and a prospective study design is required. The CU ratio using isocontouring may be sensitive to spatial image resolution and PET scanner variability. Subsequent investigations should evaluate the impact of different spatial resolutions and PET scanners, incorporating harmonization techniques (e.g., scanner-specific calibration phantoms or image postprocessing methods) and sensitivity analyses, thereby improving generalizability and clinical applicability.

Findings from the current study are restricted to ^18^F-FET–active tumors (76% of confirmed preoperative glioma with available imaging in the current study). In PET photopenic CNS cancer, advanced imaging techniques, such as MR spectroscopy, chemical exchange saturation transfer, or diffusion MRI, would certainly provide supplementary information, which should also be investigated in multimodal and multiparametric research for IDH genotyping—with potential to obviate a preceding biopsy. Furthermore, diffuse and multifocal tumor manifestations may result in divergent metabolic and molecular signatures. The current cohort comprised a mixed patient population, which is reflective of the situation in clinical practice. A study population with the same histologic subtype could provide greater comparability at the cost of restricted generalizability; nonetheless, our results suggest that the CU ratio reflects metabolic reprogramming independent of tumor entity. Of particular note is that former IDH-mutated glioblastomas are classified as astrocytoma CNS WHO grade 4 and that oligodendrogliomas are genetically defined by IDH mutation and LOH1p/19q according to the 2021 WHO classification (1). Although the determined isocontouring thresholds achieve plausible segmentation into central and peripheral metabolic compartments, an immunohistochemical correlation and further optimization of thresholds based on tissue specimens merit further research. Future studies should investigate response assessment of IDH-targeted therapy, as well as CU characteristics in other IDH mutation–associated tumors, including acute myeloid leukemia, cholangiocarcinoma, or chondrosarcoma. Multilateral interactions between cancer metabolism, oncogenic pathways, and the tumor microenvironment, particularly interactions between cancer, immune, and neuronal cells, are further areas for future studies.

## CONCLUSION

The IDH genotype has a significant impact on the diagnosis and treatment of glioma. We proposed parametric ^18^F-FET PET as a noninvasive metabolic biomarker for the classification of IDH genotype—with critical implications for clinical management and the diagnostic workup of patients with CNS cancer.

## DISCLOSURE

This research project was funded by the “Deutsche Forschungsgemeinschaft” (DFG, German Research Foundation) (SFB1340/1 2018, SFB1315, and SFB295RETUNE). The PET/MRI scanner was cofunded by the “Deutsche Forschungsgemeinschaft” (INST 335/543-1 FUGG 2015). No other potential conflict of interest relevant to this article was reported.

## Notes

KEY POINTS **QUESTION:** Is the IDH genotype—a critical regulator of glucose and amino acid metabolism—associated with a distinct metabolic phenotype in amino acid PET? **PERTINENT FINDINGS:** Fifty-two patients with preoperative glioma were retrospectively investigated using static ^18^F-FET PET. Metabolic tumor volume was distinct and presented greater dimensions than contrast enhancement, which is known to underestimate tumor margins. Evaluation of compartmental ^18^F-FET uptake characteristics determined IDH genotype with excellent diagnostic performance, establishing a critical association between spatial metabolic heterogeneity, mitochondrial tricarboxylic acid cycle, and genomic features. Molecular classification of LOH1p/19q, MGMT promoter methylation, and ATRX loss by spatial metabolic patterns was possible, suggesting indirect associations with tyrosine metabolism. **IMPLICATIONS FOR PATIENT CARE:** We proposed parametric ^18^F-FET PET as a noninvasive metabolic biomarker for the classification of IDH genotype before surgical intervention, with implications for clinical management and the diagnostic workup of patients with glioma.
